# Supplementary material for: Theoretical Prediction of Mechanical Strength and Desalination Performance of One-Atom-Thick Hydrocarbon Polymer in Pressure-Driven Separation
Source: Polymers (Basel). 2019 Aug 16;11(8):1358. doi: 10.3390/polym11081358 (PMC6723690; doi:10.3390/polym11081358)
Supplement: Supplementary file 1 [file polymers-11-01358-s001.pdf]

Supporting Information

Theoretical Prediction of Mechanical Strength and Desalination Performance of One-Atom-Thick Hydrocarbon Polymer in Pressure-Driven Separation

Shuangqing Sun,[1, 2] Fei Shan,[1] Qiang Lyu,[1] Chunling Li[1,2,*], Songqing Hu[1,2,*]

[1]School of Materials Science and Engineering, China University of Petroleum (East China), Qingdao 266580, China.

[2]Institute of Advanced Materials, China University of Petroleum (East China), Qingdao 266580, China

* Correspondence: lichunling@upc.edu.cn (C.-L.L.); songqinghu@upc.edu.cn (S.-Q.H.)

Contents:

1. Structure optimization

2. Non-bonding interaction parameters

3. Relationship between stress and substrate pore size

1. Structure optimization

The fully optimized unit cell structures and lattice parameters of two-dimensional (2D) hydrocarbon polymer membranes studied in this work are summarized in Figure S1 and Table S1, respectively. The unit cells of these structures were relaxed using van-der-Waals-corrected density functional theory (DFT) calculations. The generalized gradient approximation (GGA) with the Perdew-Burke-Ernzerhof (PBE) functional implemented in Dmol^3 was employed for the exchange-correlation energy of interacting electrons. In these calculations, a dispersion correction by the Grimme's method was also included. Double numerical plus polarization (DNP) basis sets were used to expand electronic wave functions. Self-consistent field (SCF) calculations were performed with a convergence criterion of 10^{-6} a.u. for the total energy. In addition, a real-space global orbital cutoff radius of 4.5 Å and a thermal smearing parameter of 0.002 Ha were chosen. The Brillouin zone was sampled using 6 × 6 × 1 Monkhorst-Pack meshes, and a 20 Å vacuum thickness was used to prevent the interaction of structures between periodic images. All structures were fully relaxed until the following convergence criteria were met: less than 1×10^{-5} Ha in energy, 2×10^{-3} Ha·Å^{-1} in maximum force, and 5×10^{-3} Å in maximum displacement. As given in Table S1, the optimized lattice parameters of PG are $a = b = 7.4926$ Å, which are in excellent agreement with previous theoretical and experimental studies (i.e., $a = b = 7.455$ Å or 7.53 Å from other theoretical calculations[1, 2] and $a = b = 7.4$ Å from experiments[3]).

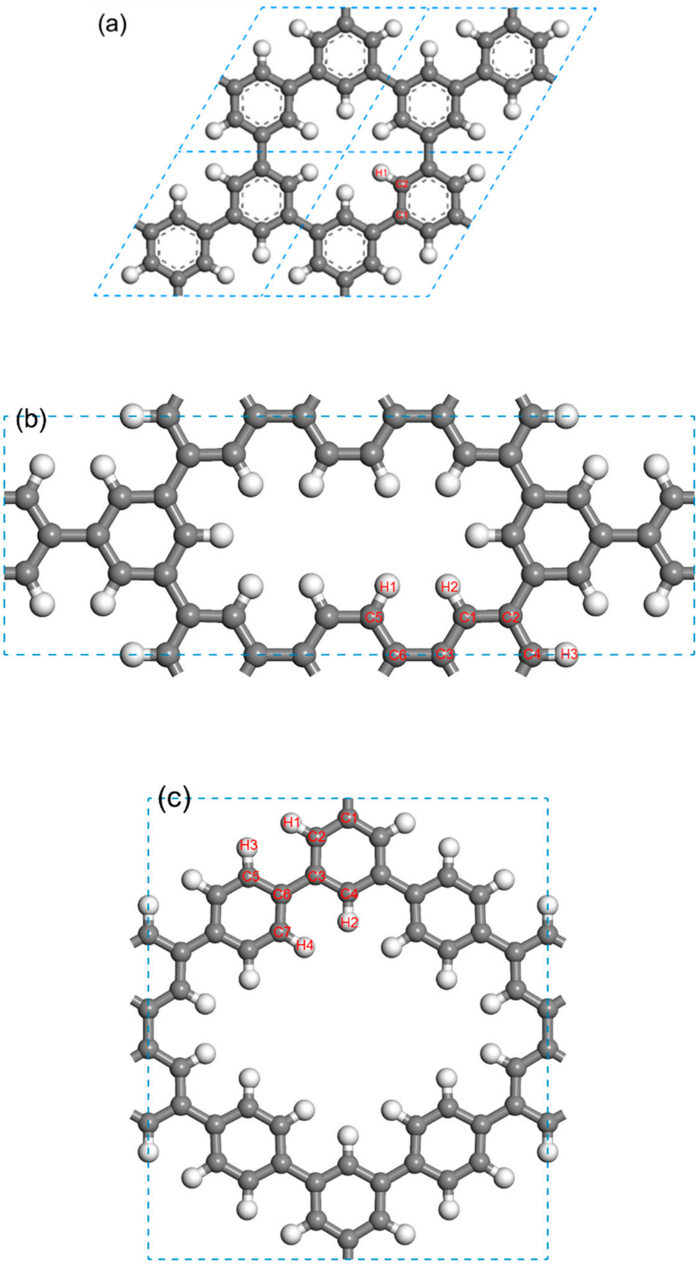
(a)
(b)
H1
H2
C5
C1
C2
C6
C3
C4
H3
(c)
H1
C1
H3
C2
C5
C3
C6
C4
H2
C7
H4

(d)

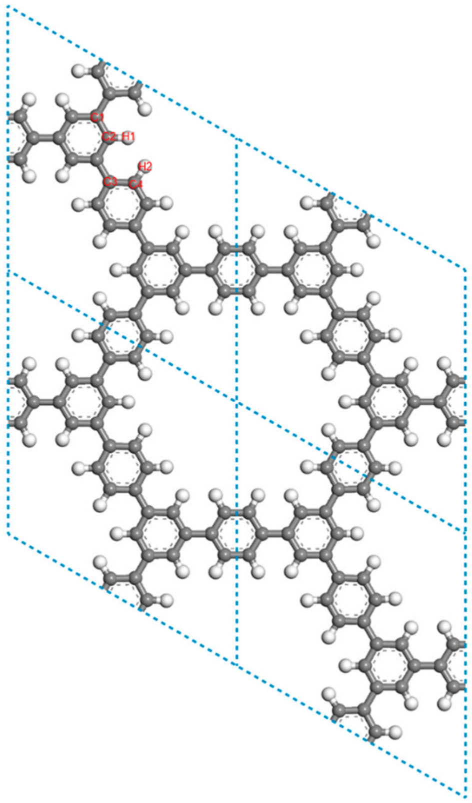

Figure S1. Optimized structures of hydrocarbon polymer membranes using DFT calculations. The corresponding pore characteristics of each structure shown in this figure can be found in Table S1. Color code: grey: carbon atoms and white: hydrogen atoms. Dashed lines are displayed to indicate the unit cell of each structure.

Table S1. Optimized lattice parameters of each studied structure as shown in Figure S1.

Structure	Space group	a (Å)	b (Å)	α (°)	β (°)	γ (°)
(a) PG	*P6/mmm*	7.4926	7.4926	90	90	120
(b) PG-TP1	*Cmmm*	21.666	7.4838	90	90	90
(c) PG-TP2	*Cmmm*	15.0349	17.350	90	90	90
(d) PG-TP3	*P6/mmm*	14.880	14.880	90	90	120

2. Non-bonding interaction parameters

Table S2. Non-bonding interaction parameters adopted in this work. Charges for atoms in the piston and solution can be found here. Charges for atoms in the studied PG structures can be found in Table S3, as noted in the table.

Element	ε (kcal/mol)	σ (Å)	q (e)	Ref.
C_{piston}	0.0565	3.2140	0.0000	[4]
$C_{membrane}$	0.0951	3.4730	**Table S3**	[5]
$H_{membrane}$	0.0152	2.8464		[5]
O_{water}	0.1553	3.1660	-0.8476	[6]
H_{water}	0.0000	0.0000	0.4238	[6]
Na^{+}	0.3526	2.1595	1.0000	[7]
Cl^{-}	0.0128	4.8305	-1.0000	[7]

Table S3. Atomic partial charges derived from DFT calculations for atoms in studied PG structures. The definition of atom types for each structure can be found in Figure S1.

**Structure**	q_{C1} **(e)**	q_{C2} **(e)**	q_{C3} **(e)**	q_{C4} **(e)**	q_{C5} **(e)**	q_{C6} **(e)**	q_{C7} **(e)**	q_{H1} **(e)**	q_{H2} **(e)**	q_{H3} **(e)**	q_{H4} **(e)**
**(a) PG**	-0.0009	-0.0606						0.0614			
**(b) PG-TP1**	-0.0828	0.0187	0.0166	-0.0816	- 0.0902	0.0418		0.0707	0.0629	0.0646	
**(c) PG-TP2**	0.0185	-0.0828	0.0171078	-0.0842	-0.0907	0.0421	-0.0908	0.0643	0.0638	0.0708	0.0714
**(d) PG-TP3**	0.0173	-0.0840	0.0422	-0.0909				0.0640	0.0713		

3. Stress as a function of substrate pore radius

The curves of PG, PG-TP1 and PG-TP3 are shown below. When the external pressure reaches 5 MPa, the membrane does not break with pore of substrate of PG less than 5 μm. It can be seen from the Figure S2 that when the external pressure reaches 5 MPa and the pore size of substrate of PG-TP1 reaches about 2.6 μm, the stress reaches the tearing stress, and material will break. The pore size of substrate of PG-TP3 need less than 2.9 μm.

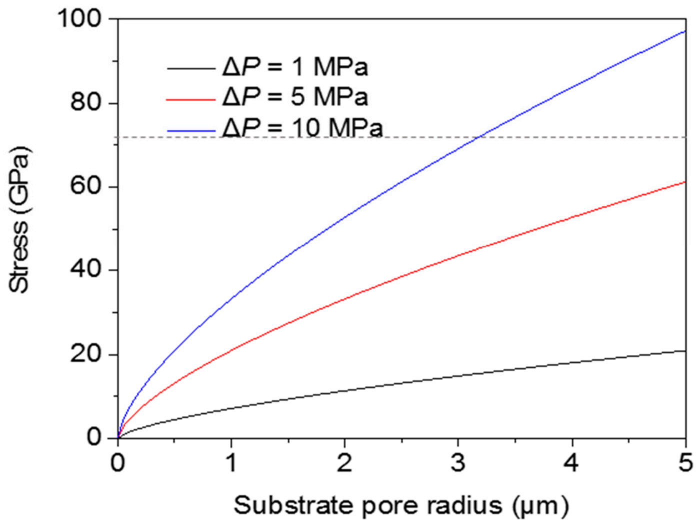

Figure S2. Stress as a function of substrate pore radius (PG).

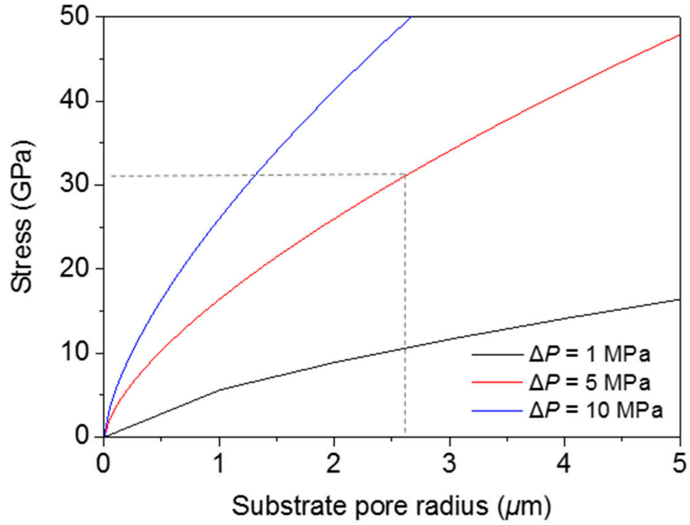

Figure S3. Stress as a function of substrate pore radius (PG-TP1).

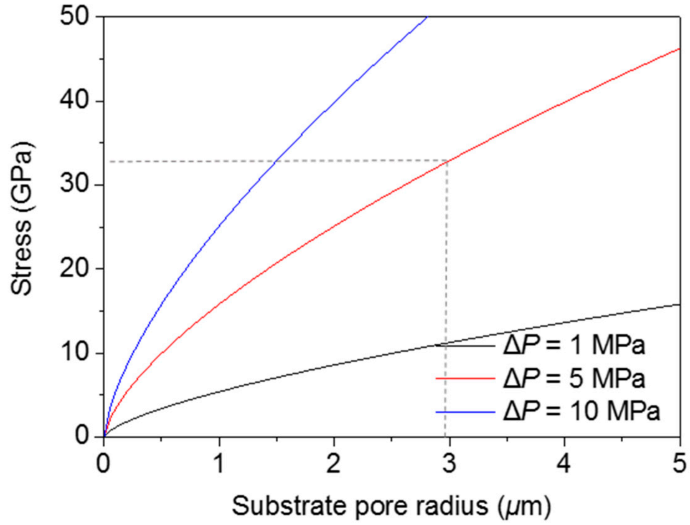

Figure S4. Stress as a function of substrate pore radius (PG-TP3).

References:

1. Li, Y.; Zhou, Z.; Shen, P.; Chen, Z. Two-dimensional polyphenylene: experimentally available porous graphene as a hydrogen purification membrane, *Chem. Commun.* **2010**, *46*, 3672-3674.
2. Wei, S.; Zhou, S.; Wu, Z.; Wang, M.; Wang, Z.; Guo, W.; Lu, X. Mechanistic insights into porous graphene membranes for helium separation and hydrogen purification, *Appl. Surf. Sci.* **2018**, *441*, 631-638.
3. Bieri, M.; Treier, M.; Cai, J.; Aït-Mansour, K.; Ruffieux, P.; Gröning, O.; Gröning, P.; Kastler, M.; Rieger, R.; Feng, X. Porous graphenes: two-dimensional polymer synthesis with atomic precision, *Chem. Commun.* **2009**, 6919-6921.
4. Werder, T.; Walther, J. H.; Jaffe, R. L.; Halicioglu, T.; Koumoutsakos, P. On the Water−Carbon Interaction for Use in Molecular Dynamics Simulations of Graphite and Carbon Nanotubes, *J. Phys. Chem. B*, *112*, 14090-14090.
5. Mayo, S. L.; Olafson, B. D.; Goddard, W. A. DREIDING: a generic force field for molecular simulations, *J. Phys. Chem.* **1990**, *94*, 8897-8909.
6. Berendsen, H. J. C.; Grigera, J. R.; Straatsma, T. P. The missing term in effective pair potentials, *J. Phys. Chem.* **1987**, *91*, 6269-6271.
7. Joung, I. S.; Cheatham, Thomas E. Determination of Alkali and Halide Monovalent Ion Parameters for Use in Explicitly Solvated Biomolecular Simulations, *J. Phys. Chem. B* **2008**, *112*, 9020.
